# Mycophenolate Antagonizes IFN-γ-Induced Catagen-Like Changes via β-Catenin Activation in Human Dermal Papilla Cells and Hair Follicles

**DOI:** 10.3390/ijms150916800

**Published:** 2014-09-22

**Authors:** Sunhyo Ryu, Yonghee Lee, Moo Yeol Hyun, Sun Young Choi, Kwan Ho Jeong, Young Min Park, Hoon Kang, Kui Young Park, Cheryl A. Armstrong, Andrew Johnson, Peter I. Song, Beom Joon Kim

**Affiliations:** 1 Department of Dermatology, Chung-Ang University College of Medicine, Seoul 137-701, Korea; E-Mails: bktsh0328@gmail.com (S.R.); iamyonghee84@naver.com (Y.L.); drhmy@naver.com (M.Y.H.); sun02ya@naver.com (S.Y.C.); momo920@daum.net (K.Y.P.); 2 Department of Medicine, the Graduate School, Chung-Ang University, Seoul 137-701, Korea; 3 Department of Dermatology, St. Paul’s Hospital, College of Medicine, the Catholic University of Korea, Seoul 137-701, Korea; E-Mails: kwanho16@nate.com (K.H.J.); johnkang@catholic.ac.kr (H.K.); 4 Department of Dermatology, Seoul St. Mary’s Hospital, College of Medicine, the Catholic University of Korea, Seoul 137-701, Korea; E-Mail: yymmpark6301@hotmail.com; 5 Department of Dermatology, Denver Health Medical Center, Denver, CO 80204, USA; E-Mail: Cheryl.Armstrong@dhha.org; 6 Department of Dermatology, University of Arkansas for Medical Sciences, Little Rock, AR 72205, USA; E-Mail: AJohnson4@uams.edu; 7 Department of Dermatology, University of Colorado Denver Anschutz Medical Campus, Aurora, CO 80045, USA; E-Mail: peter.song@ucdenver.edu

**Keywords:** MPA, β-catenin, GSK3β, DKK-1, IFN-γ, TGF-β2, dermal papilla cell, hair growth

## Abstract

Recently, various immunosuppressant drugs have been shown to induce hair growth in normal hair as well as in alopecia areata and androgenic alopecia; however, the responsible mechanism has not yet been fully elucidated. In this study, we investigate the influence of mycophenolate (MPA), an immunosuppressant, on the proliferation of human dermal papilla cells (hDPCs) and on the growth of human hair follicles following catagen induction with interferon (IFN)-γ. IFN-γ was found to reduce β-catenin, an activator of hair follicle growth, and activate glycogen synthase kinase (GSK)-3β, and enhance expression of the Wnt inhibitor DKK-1 and catagen inducer transforming growth factor (TGF)-β2. IFN-γ inhibited expression of ALP and other dermal papillar cells (DPCs) markers such as Axin2, IGF-1, and FGF 7 and 10. MPA increased β-catenin in IFN-γ-treated hDPCs leading to its nuclear accumulation via inhibition of GSK3β and reduction of DKK-1. Furthermore, MPA significantly increased expression of ALP and other DPC marker genes but inhibited expression of TGF-β2. Therefore, we demonstrate for the first time that IFN-γ induces catagen-like changes in hDPCs and in hair follicles via inhibition of Wnt/β-catenin signaling, and that MPA stabilizes β-catenin by inhibiting GSK3β leading to increased β-catenin target gene and DP signature gene expression, which may, in part, counteract IFN-γ-induced catagen in hDPCs.

## 1. Introduction

A hair follicle (HF) is an organ composed of epithelial and mesenchymal tissues. The post-natal hair follicle undergoes a cycle of growth (anagen), regression (catagen) and rest (telogen) [1]. The reciprocal interactions between epithelial and mesenchymal tissues are essential for the growth and development of HFs [2,3]. The dermal papilla (DP) is a cluster of specialized fibroblasts enveloped by hair matrix keratinocytes in the bulb of anagen hair that activates the keratinocytes to maintain and regenerate the hair growth cycle [4]. Moreover, effective interaction between the DP and surrounding epithelial cells is essential to form new HFs [5]. Therefore, dermal papillar cells (DPCs) play a decisive role in the regulation of hair growth and reconstitution of HFs.

Interferon (IFN)-γ is among the growing number of cytokines involved in the regulation of HFs cycling [4,6,7,8]. For example, both strains of transgenic mice that overexpress IFN-γ exhibit hair loss [9,10]. Recent studies suggested that IFN-γ is a potent catagen inducer in normal human scalp HFs, which express IFN-γRβ in both the hair matrix and in the DP [11]. Catagen induction by IFN-γ probably occurs, at least in part, via upregulation of the recognized catagen-stimulatory growth factor TGF-β2 [11]. However, the underlying mechanisms of IFN-γ-induced catagen in human dermal papilla cells (hDPCs) and in HFs are not completely understood. In this study, our first aim was to investigate the mechanism of catagen induction in hDPCs using IFN-γ treatment *in vitro*.

The Wnt/β-catenin pathway is one of the most important elements of hair growth regulation. β-catenin is markedly activated in DPCs during anagen [12,13] and is important for regeneration of the hair cycle [14,15]. Premature induction of catagen and telogen leading to failure of follicle regeneration has been observed in β-catenin knockout mice, indicating that activation of β-catenin in DPCs can prolong the anagen phase and promote hair growth [16].

Mycophenolate (MPA) is an immunosuppressant drug used to prevent rejection in organ transplantation. Recent studies have demonstrated stimulation of hair growth by immunosuppressants such as cyclosporin A (CsA) and tacrolimus [17,18]. Repeated topical application of CsA is an effective treatment for alopecia areata in humans and rats, and oral administration promotes hair growth in athymic nude mice [19,20,21,22,23]. Topical application of tacrolimus onto the skin of mice, rats, and hamsters markedly stimulates hair growth, and this effect may be unrelated to its immunosuppressive properties [24]. A similar effect of MPA on hair growth stimulation has not yet been directly examined, and thus the mechanism for this effect remains to be elucidated.

The second aim of this study was to investigate the effects of MPA on human hair growth *in vitro* using cultured hDPCs and organ cultures of human hair follicles with catagen induced by IFN-γ treatment in both. We investigated which signaling pathways mediate IFN-γ catagen induction in hDPCs. We then studied how MPA antagonizes the IFN-γ-induced catagen-like changes in these cells. Since β-catenin activity in DPCs is implicated in mediating anagen duration, we investigated whether IFN-γ inhibits the β-catenin pathway and whether MPA activates the β-catenin pathway after catagen induction by IFN-γ in hDPCs. Finally, we investigated the effect of MPA on human HFs cultured *ex vivo* that were treated with IFN-γ to induce catagen-like changes.

## 2. Results and Discussion

### 2.1. MPA Enhances the Proliferation of Human Dermal Papilla Cells (hDPCs)

**Figure 1:**
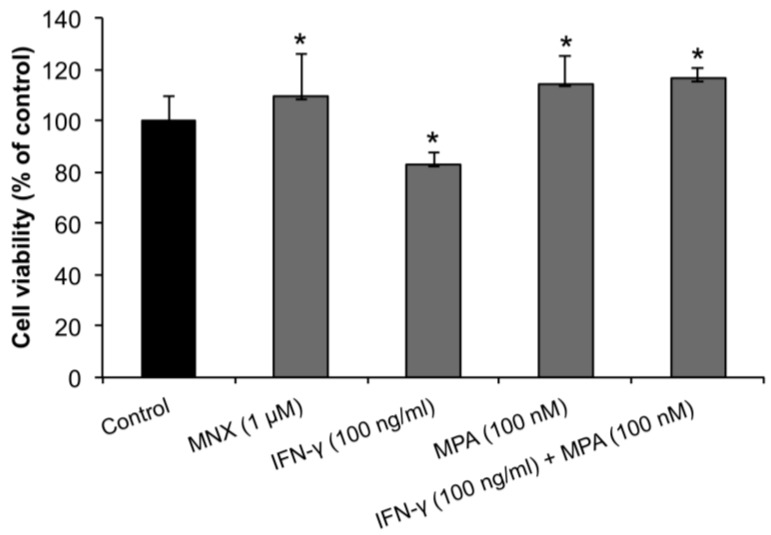
Mycophenolate (MPA) promotes human dermal papilla cell (hDPC) proliferation and abrogates IFN-γ-mediated hDPC reduction. hDPCs were treated with 100 ng/mL of IFN-γ, 100 nM of MPA, or with both IFN-γ and MPA for 3 days, and MTT assay was assessed on day 3. MNX served as a positive control for hDPC promotion. Results were expressed as mean ± SEM of percent change compared to the control. Statistically significant differences were determined by ANOVA (* *p* < 0.05).

In order to determine the effect of MPA on proliferation of hDPCs we performed the MTT assay 3 days after culture in the presence or absence of IFN-γ. As shown in Figure 1, MPA significantly enhanced the proliferation of hDPCs compared to untreated negative controls and minoxidil (MNX, an activator of β-catenin pathway in hDPCs)-treated positive controls [25] with optimal enhancement at 100 nM. Treatment with IFN-γ (100 ng/mL) significantly inhibited the proliferation of hDPCs compared to the MNX-treated positive control. MPA significantly counteracted the inhibitory effect of IFN-γ on hDPC proliferation.

### 2.2. Mycophenolate (MPA) Activates the β-Catenin Pathway in Interferon (IFN)-γ-Treated hDPCs.

**Figure 2:**
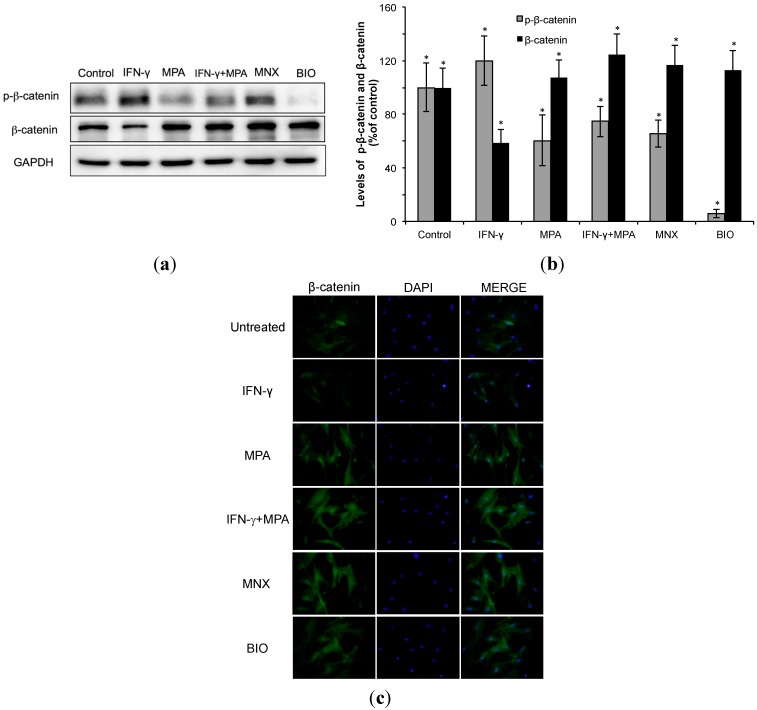
Stabilization and nuclear accumulation of β-catenin in hDPCs treated with MPA. hDPCs were treated with 100 ng/mL of IFN-γ, 100 nM of MPA, or with both IFN-γ and MPA for 48 h. (**a**) The protein level of β-catenin was examined by Western blot using anti-phospho-β-catenin and anti-β-catenin antibodies; (**b**) The relative level of β-catenin protein was shown as mean ± SD from three independent experiments. Statistically significant differences were determined by ANOVA (* *p* < 0.05); and (**c**) Intracellular localization of β-catenin was determined by immunocytochemistry staining using specific anti-β-catenin antibodies (green). Corresponding DAPI nuclear staining (blue) and the merged images are also shown. minoxidil (MNX) and 6-bromoindirubin-30-oxime (BIO) served as positive controls for stabilization and nuclear translocation of β-catenin.

We examined whether β-catenin signaling is regulated by MPA in hDPCs. As shown in Figure 2a,b, IFN-γ reduced the total level of β-catenin protein and increased phopho-β-catenin protein in hDPCs compared to all other treatments and controls. Both minoxidil (MNX) and the GSK3β inhibitor, 6-bromoindirubin-30-oxime (BIO) increased the total amount of β-catenin protein and decreased the level of phospho-β-catenin protein. Treatment with MPA for 24 h reduced the level of inactive phospho-β-catenin and increased the total amount of β-catenin protein in cultured hDPCs. Furthermore, MPA counteracted the reduction of β-catenin by IFN-γ and also limited the abundance of phosphor-β-catenin protein seen with IFN-γ treatment alone.

Next, cellular localization of β-catenin was examined after treating hDPCs for 24 h with MPA alone, IFN-γ alone, or MPA and IFN-γ together, with MNX as a positive control. In IFN-γ-treated cells, β-catenin was very weakly detected in the nuclei. However, MPA (100 nM) induced nuclear translocation of β-catenin in hDPCs to a similar extent as the positive control 1 µM MNX (Figure 2c).

### 2.3. MPA Activates the β-Catenin Pathway by Inhibition of GSK3β and Reduction of DKK-1 Expression in hDPCs

**Figure 3:**
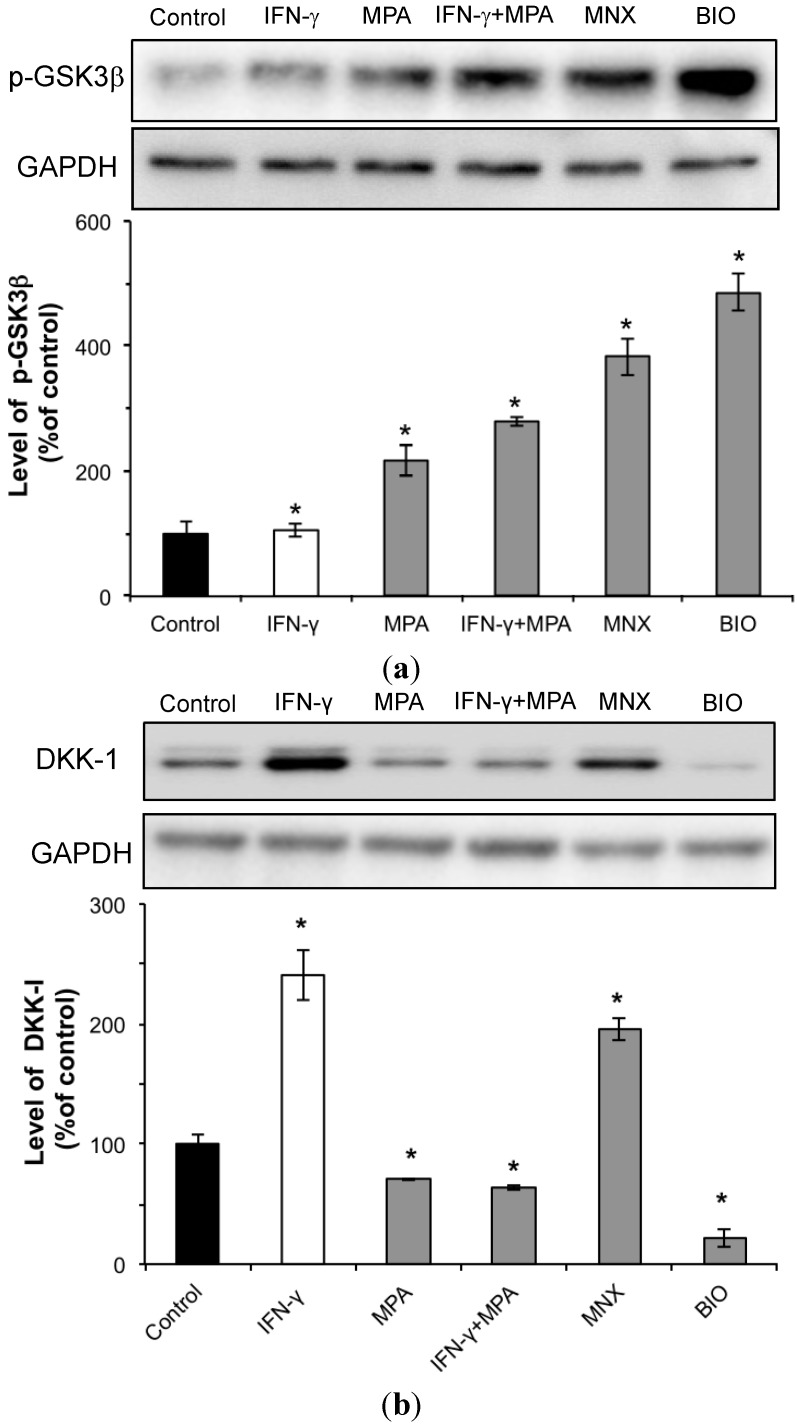
Phosphorylation of GSK3β and expression of DKK-1 in hDPCs following treatment with MPA and/or IFN-γ. hDPCs were treated with 100 ng/mL of IFN-γ, 100 nM of MPA, or with both IFN-γ and MPA for 48 h. The levels of phospho-GSK3β (**a**) and DKK-1 (**b**) were examined by Western blot using anti-phospho-GSK3β or anti-DKK-1 antibodies, respectively. Each relative protein level of phospho-GSK3β and DKK-1 is also shown as mean ± SD from three independent experiments. Statistically significant differences were determined by ANOVA (* *p* < 0.05). MNX and BIO served as positive controls for phosphorylation of GSK3β and expression of DKK-1.

GSK3β is an upstream effector of β-catenin. As shown in Figure 3a, MPA increased the amount of phosphorylated GSK3β, the inactive form of GSK3β, leading to activation of the β-catenin pathway. IFN-γ significantly reduced phospho-GSK3β in hDPCs, whereas MPA increased phospho-GSK3β after IFN-γ treatment. BIO (1 µM) was used as a positive control for detection of phospho-GSK3β. The total GSK3β protein level remained constant in all cells (data not shown). These results suggest that GSK3β inhibition via phosphorylation is the likely mechanism responsible for increased β-catenin protein following treatment with MPA in IFN-γ-treated hDPCs, as opposed to increased β-catenin synthesis.

Since Dickkopf (DKK)-1 promotes regression of hair follicles by blocking Wnt/β-catenin signaling [26], we investigated the expression of DKK-1 in hDPCs treated with MPA and/or IFN-γ. IFN-γ significantly increased the amount of DKK-1 in hDPCs, and MPA co-treatment completely abolished this IFN-γ-dependent increase of DKK-1 in hDPCs (Figure 3b).

### 2.4. MPA Regulates the Expression of β-Catenin Pathway Target Genes and Dermal Papilla (DP) Signature Genes in IFN-γ-Treated hDPCs

**Figure 4:**
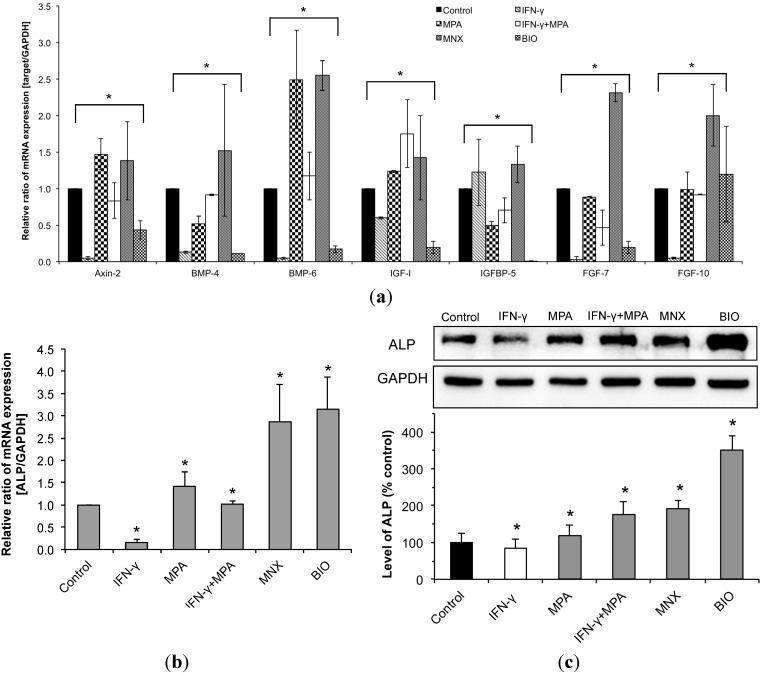
Effects of MPA on the expression of β-catenin target genes, DP signature genes, and alkaline phosphatase (ALP) in IFN-γ-treated hDPCs. mRNA expression was measured by real-time RT-PCR using appropriate primers, as described in “Materials and Methods”. The relative intensity of each mRNA expression was normalized with GAPDH. mRNA expression of DP signature genes (**a**), β-catenin target genes (**a**), and ALP (**b**) are shown; and (**c**) The protein level of ALP was examined by Western blot using a specific anti-ALP antibody, and its relative level is shown as mean ± SD from three independent experiments. MNX and BIO served as positive controls for expression of DP signature genes, β-catenin target genes, and ALP. Statistically significant differences were determined by ANOVA (* *p* < 0.05).

We examined changes in the expression of β-catenin target genes and DP signature genes by real-time PCR using hDPCs treated with MPA and/or IFN-γ for 24 h. Expression of Axin-2 was used as an indicator of β-catenin signaling pathway activity, which promotes the phosphorylation and degradation of β-catenin [27]. In hDPCs IFN-γ significantly reduced Axin-2 expression whereas MPA enhanced Axin-2 expression despite co-treatment with IFN-γ (Figure 4a). When we examined the expression of DP signature genes such as bone morphogenic proteins (BMPs), fibroblast growth factors (FGFs), and insulin-like growth factor (IGF)-1 [28], we found that IFN-γ significantly inhibited the expression of BMP4, BMP6, IGF-1 and FGF7; however, MPA strongly enhanced the expression of these genes in hDPCs despite the presence of IFN-γ (Figure 4a). Moreover, IFN-γ significantly inhibited the expression of alkaline phosphatase (ALP), the activity of which is related to follicle induction [29]. Inhibition of ALP expression by IFN-γ at both the mRNA and protein level was blocked by MPA (Figure 4b,c), and MPA alone also significantly enhanced ALP expression in hDPCs. These results suggest that MPA may enhance hDPC stimulation of HFs growth despite the presence of IFN-γ, a strong catagen inducer.

### 2.5. MPA Inhibits the Up-Regulation of Transforming Growth Factor (TGF)-β2 Induced by IFN-γ in hDPCs.

**Figure 5:**
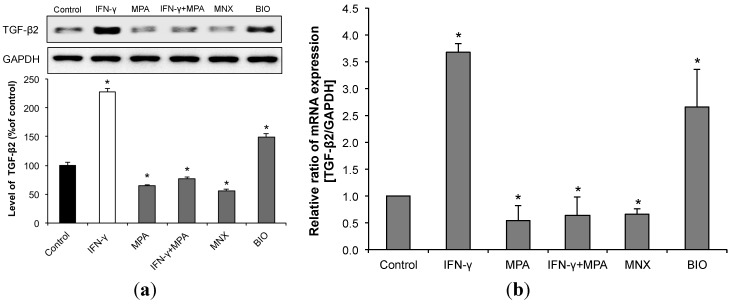
MPA reduces expression of the catagen-inducing cytokine TGF-β2 in hDPCs even following treatment with IFN-γ. hDPCs were treated with 100 ng/mL of IFN-γ, 100 nM of MPA, or with both IFN-γ and MPA for 48 h. (**a**) The protein level of TGF-β2 was examined by Western blot using an anti-TGF-β2 antibody, and its relative level was normalized to that of GAPDH; (**b**) TGF-β2 mRNA expression was measured by real-time RT-PCR using its specific sense and antisense primers, as described in “Materials and Methods”. The relative expression of each mRNA was normalized to that of GAPDH. MNX and BIO served as controls for TGF-β2 expression. Results were expressed as mean ± SD from three independent experiments. Statistically significant differences were determined by ANOVA (* *p* < 0.05)*.*

Since IFN-γ significantly enhances *TGF-β2* gene and protein expression in human HFs suggesting that IFN-γ induces catagen, at least in part, via up-regulation of TGF-β2 [11], we examined the expression of TGF-β2 in hDPCs treated with IFN-γ and MPA. We found that IFN-γ significantly enhanced TGF-β2 expression in hDPCs at both the protein and mRNA level, and that MPA strongly inhibited this induction of TGF-β2 (Figure 5a,b). These results suggest that MPA antagonizes IFN-γ-induced catagen-like changes, in part, through inhibition of TGF-β2 expression in hDPCs.

### 2.6. MPA Abrogates IFN-γ Inhibition of Hair Growth ex Vivo in a Culture Model

**Figure 6:**
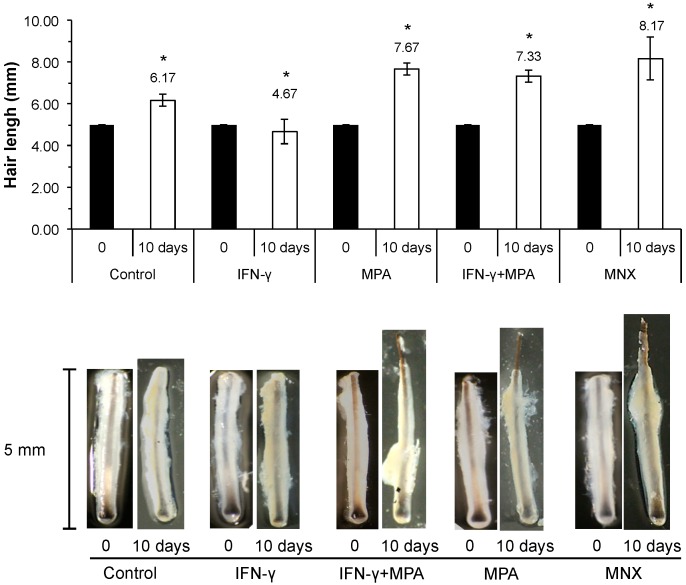
MPA stimulates growth IFN-γ-treated hair follicles in organ culture. A total of 350 human scalp hair follicles with intact dermal papillae were obtained and treated for 10 days with IFN-γ (100 ng/mL), MPA (100 nM), or both IFN-γ and MPA. The length of each hair follicle was measured under a microscope on days 0 and 10. The relative length of each hair shaft is shown as mean ± SD (**upper panel**). Images of representative hair follicles at days 0 and 10 are shown in the **lower panel**. MNX (1 µM) served as positive controls for stimulation of hair follicle growth. All values were expressed as mean ± SD. Statistically significant differences were determined by ANOVA (* *p* < 0.05).

In order to examine the effect of MPA in the presence or absence of IFN-γ at the organ level, we performed *ex vivo* culture of whole human scalp HFs. In this study, 350 HFs from three different individuals were cultured with MPA, IFN-γ or MPA plus IFN-γ for 10 days. Elongation of each hair shaft was measured every third day. MNX and vehicle served as positive and negative controls, respectively. As shown in Figure 6, HFs treated with MPA (100 nM) grew longer than negative control HFs at 10 days, which was similar to the growth of HFs treated with MNX (1.0 µM), which is known to stimulate hair growth. Low-dose IFN-γ (less than 100 ng/mL) produced no significant impairment of hair shaft elongation compared to vehicle (data not shown), but at the dose of 100 ng/mL IFN-γ significantly inhibited hair shaft elongation. We observed a narrower hair bulb in HFs treated with IFN-γ at the 100 ng/mL dose, which is reminiscent of catagen-like regressive changes. Thus, IFN-γ treatment at the dose of 100 ng/mL was used to establish an *ex vivo* model of HFs catagen induction. With co-incubation of MPA (100 nM) and IFN-γ (100 ng/mL), MPA significantly abrogated IFN-γ-induced growth inhibition of cultured HFs *ex vivo*.

### 2.7. Discussion

Various immunosuppressant medications have been shown to stimulate hair growth in normal hair and in conditions of abnormal hair growth such as alopecia areata and androgenic alopecia. Paus *et al.* showed that CsA at high doses induces anagen in resting (*i.e.*, telogen) follicles [20], and inhibits pharmacologically-triggered massive catagen development [30] and chemotherapy-induced alopecia in mice [31]. High doses of CsA and tacrolimus significantly induce anagen and disrupt the spontaneous transition to catagen [18]. Furthermore, Gafter-Gvili *et al.* reported that calcineurin activity is associated with hair keratinocyte differentiation *in vivo*, affecting nuclear factor of activated T cells (NFAT1) activity [32]. Treatment of nude or C57BL/6 depilated normal mice with CsA inhibits the expression of keratinocyte terminal differentiation markers associated with catagen, along with the inhibition of calcineurin and NFAT1 nuclear translocation. This is associated with induction of hair growth in nude mice and retardation of spontaneous catagen induction in depilated normal mice. Thus, calcineurin may be functionally active in follicular keratinocytes and that inhibition of the calcineurin-NFAT1 pathway in these cells* in vivo* by CsA enhances hair growth [32]. However, the more exact mechanisms of hair growth stimulation by immunosuppressant medications remain to be elucidated. In this study, we demonstrate that mycophenolate prolongs the anagen phase of HFs through direct interaction with DPCs, providing the first direct evidence that an immunosuppressant medication has an essential regulatory role in the control of hair growth. Our results suggest that MPA maintains anagen-phase characteristics in DPCs and blocks the IFN-γ-induced catagen transition of human HFs through activation of the Wnt/β-catenin signaling pathway. This is consistent with the recently increasing recognition that Wnt/β-catenin signaling is crucial for murine hair growth [3,15,33,34,35,36,37,38].

In this study, we demonstrated that MPA increases the expression of β-catenin target genes and some DP signature genes such as ALP, Axin2, IGF-1, and FGF7. We also demonstrated that MPA suppresses TGF-β2, which has been known to be a potent catagen promoting factor in murine [39] and human HFs [8,40,41,42,43]. Ito* et al.* previously reported that IFN-γ up-regulates TGF-β2 in the lower hair bulb, suggesting that the catagen induction by IFN-γ may, at least in part, be due to up-regulation of other potent catagen inducers such as TGF-β2 [11]. In this study, we demonstrated that MPA efficiently inhibits IFN-γ-induced TGF-β2 expression in hDPCs, which may be a significant mechanism for MPA abrogation of IFN-γ-mediated catagen induction.

Enshell-Seijffers* et al.* reported that deletion of the *β-catenin* gene in DPCs results in reduced proliferation of matrix keratinocytes surrounding the DP, thus fewer progeny cells that generate the hair shaft, and also reported that inactivation of β-catenin within the DPCs of fully developed HFs causes premature induction of the catagen phase of the hair cycle in mice [16]. Wnt/β-catenin signaling activation by inhibiting GSK3β maintains the ability of hDPCs to induce hair growth, and β-catenin activity in the DP regulates morphogenesis and regeneration of hair [16,44]. In this study, we demonstrate for the first time that IFN-γ activates GSK3β to inhibits β-catenin activity in DPCs, which induces catagen-like changes in these cells.

In the absence of Wnt, β-catenin is ordinarily phosphorylated in the cytoplasm by glycogen synthase kinase (GSK)-3, a serine/threonine protein kinase encoded by GSK-3α and β, and the resulting phospho-β-catenin is tagged by ubiquitin and then destroyed by the proteasome. However, when the Wnt protein binds to cell surface receptors of the Frizzled family, GSK3β is inhibited and a pool of cytoplasmic β-catenin stabilizes leading to its translocation into the nucleus resulting in transcription of target genes. Recent studies have shown that the GSK3β inhibitor BIO restores the expression of ALP and IGF-1, which are markers for hair follicle induction in DPCs, leading to support of the hair bulb and promotion of hair growth [45]. Since the β-catenin-dependent expression of genes based on Wnt signaling is detected in developing dermal condensates and in HFs [46] and strong nuclear β-catenin expression in DPCs is detected during the anagen phase [13], the nuclear accumulation of β-catenin in DPCs is important for the regeneration of follicles and hair growth. Recent studies have demonstrated that valproic acid (VPA) and other GSK3β inhibitors such as 4-phenyl butyric acid, LiCl, and BeCl_2_ activate the Wnt/β-catenin pathway* in vitro* and* in vivo* to promote hair growth [47,48,49]. Thus, GSK3 is a key protein in the regulation of Wnt/β-catenin signaling, and inhibition of GSK-3 therefore activates the Wnt/β-catenin signaling pathway to enhance promotion of hair growth by increasing active β-catenin levels in DPCs. In this study, we demonstrate that mycophenolate stabilizes β-catenin by inhibiting GSK3 in DPCs leading to increased expression of β-catenin target genes and of some DP signature genes such as Axin2, ALP, IFG-1, and FGF-2, which may counteract IFN-γ-induced catagen-like changes. Further studies are necessary to clarify the mechanistic role of elevated levels of IFN-γ in lesions of alopecia areata, but our findings suggest that MPA may counteract IFN-γ-mediated pathologic hair loss and could be a promising therapeutic agent for hair restoration.

Andl* et al.* reported that mice expressing high levels of DKK-1 in the skin display an early and complete block in the development of HFs, suggesting an inhibitory role of DKK-1 on hair growth [32]. Kwack *et al.* reported that DKK-1 is inducible by dihydrotestosterone (DHT) and that the level of DKK-1 is elevated in the scalp of patients with male pattern baldness compared to normal [50], suggesting that DKK-1 is involved in DHT-mediated balding in androgenic alopecia. They also discovered that DKK-1 is highly expressed during the anagen-to-catagen transition and suggested that DKK-1 promotes regression of HFs by blocking Wnt/β-catenin signaling and by inducing apoptosis in follicular keratinocytes [51]. Interestingly, we found that IFN-γ significantly increases the expression of DKK-1 in DPCs and may block Wnt/β-catenin signaling via induction of DKK-1. We also demonstrated that MPA efficiently suppresses IFN-γ-induced DKK-1 expression in DPCs. Therefore, MPA antagonizes IFN-γ-induced catagen-like changes in DPCs through activation of Wnt/β-catenin signaling via reduction of DKK-1. These novel findings in this study suggest that MPA may counteract DHT-mediated DKK-1 induction by reducing the level of DKK-1 in DPCs. Therefore, we suggest that MPA should be further investigated for its potential to slow, stop, or reverse hair-loss in androgenic alopecia.

## 3. Experimental Section

### 3.1. Materials

MPA was synthesized at IKSU CO., LTD. (Seoul, Korea). 3-(4,5-dimethylthiazol-2-yl)-2,5-diphenyltetrazolium bromide, dimethyl sulfixide (DMSO), MNX, and GSK3 inhibitor BIO ((2'*Z*,3'*E*)-6-bromoindirubin-3'-oxime) were purchased from Sigma Chemical Co. (St. Louis, MO, USA). Human IFN-γ was purchased from PEPERTECH (Rocky Hill, NJ, USA).

### 3.2. Cell Cultures of hDPCs

hDPCs were purchased from CEFOBIO (Seoul, Korea). Cultured hDPCs of early passage were used for experiments, and were maintained at 37 °C in a humidified atmosphere with 5% CO_2_.

### 3.3. MTT Assay

Cell viability was determined using a MTT assay that was performed by a slight modification of the method described by Wasserman *et al* [52]. Briefly, hDPCs were seeded at a density of 2 × 10^4^ cells/well into 96-well plates and cultured for 24 h. The cells were then treated with MPA, IFN-γ, or MNX for 24 or 96 h. The samples were assessed by measuring absorbance at 540 nm with an ELISA plate reader (Thermomax, Molecular Devices, Sunnyvale, CA, USA).

### 3.4. Western Blot

Each cell lysate containing 20 µg of protein was analyzed by Western blotting using the appropriate antibodies to detect protein expression. Antibodies specific to GAPDH and ALP were purchased from Santa Cruz Biotechnology, Inc. (Santa Cruz, CA, USA). DKK-1 and p-GSK3β were purchased from Abcam Inc. (Cambridge, MA, USA). Antibodies against phospho-β-catenin and β-catenin were purchased from Cell Signaling Technology (Beverly, MA, USA). Western blot was detected by chemoluminescence (Amersham Pharmacia Biotech, Piscataway, NJ, USA).

### 3.5. Real-Time RT-PCR

Real-time PCR was performed with a C1000™ Thermal Cycler (Bio-Rad, Hercules, CA, USA) using SYBR Green (Takara, Shiga, Japan). β-catenin, Axin-2, BMP-4, BMP-6, IGF-I, IGFBP-5, FGF-7, FGF-10, TGFβ2, ALP and GAPDH oligonucleotide primers were obtained from Bioneer (Seoul, South Korea) and Cosmo Genetech Co, Ltd. (Seoul, South Korea). The primer sets used were as follow: Axin-2 sense, GCAACTCAGTAACAGCCCGA; Axin-2 antisense, AAGTCAGCAGGGGCTCATCT; BMP-4 sense, CACTGGCTGACCACCTCAAC; BMP-4 antisense, GGCACCCACA-TCCCTCTACT; BMP-6 sense, AACCAACCACGCGATTGTG; BMP-6 antisense, AAGTCTCATCGTCCCACCTC; IGF-I sense, CTCTTCTACCTGGCGCTGTG; IGF-I antisense, CATACCCTGTGGGCTTGTTG; IGFBP-5 sense, AGCAAGTCAAGATCGA-GAGAGA; IGFBP-5 antisense, TTCTTTCTGCGGTCCTTCTTCA; FGF-7 sense, TTGTGGCAATCAAAGGGGTG; FGF-7 antisense, CCTCCGTTGTGTGTCC-ATTTAGC; FGF-10 sense, TTCAAGGAGATGTCCGCTGG; FGF-10 antisense, GATGCTGTACGG-GCAGTTCT; ALP sense, CAAACCGAGATACAAGCACTCCC; ALP antisense, CGAAGAGACCCAATAGGTAGTCCAC; GAPDH sense, TGGGTGTGAACCATGAGAAG; and GAPDH antisense, GCT-AAGCAGTTGGTGGTGC.

### 3.6. Immunocytochemistry Assay

hDPCs at 1 × 10^4^ cells/500 µL were seeded into chamber slides, serum-starved for 24 h, and then treated with MPA, IFN-γ, and MNX for 48 h. These cells were then fixed in 4% paraformaldehyde for 30 min. After washing with DPBS, the cells were permeabilized with 0.1% Triton-X100 in PBS for 10 min at room temperature and then blocked with 5% BSA in 0.05% Triton X-100 for 30 min at room temperature. The samples were incubated with β-catenin antibody (1:200 dilution) at 4 °C overnight. They were then washed two times with PBS and four times with distilled water followed by incubation with a FITC-conjugated secondary goat anti-mouse IgG (1:200 dilution) in 5% BSA blocking solution for 2 h at room temperature. All samples were counterstained with 4'6-diamidino-2-phenylindole (DAPI) to visualize the nuclei. Representative images were taken with a microscope (Olympus Optical, Tokyo, Japan) at 400×.

### 3.7. Hair Follicles Organ Culture and Assessment of Hair Elongation

Anagen HFs from human scalp skin specimens was obtained. The medical ethical committee of the Chung-Ang University approved specimen collection (IRB 14-0004, 30 April 2014). Four hair follicles per well in 24-well plates were cultured in Williams E medium at 37 °C in a humidified atmosphere with 5% CO_2_ in 500 μL of basal medium supplemented with insulin 10 μg/mL, hydrocortisone 10 ng/mL, streptomycin 10 μg/mL, and penicillin 100 U/mL according to the Philpott’s method as described [53]. Each experimental group contained at least 60 anagen HFs derived from three different human (donors/volunteers). IFN-γ (100 ng/mL) was determined to optimally induce catagen-like changes, shorten hair shaft length, and hair bulb with minimal other histological alterations of the HFs. MPA or MNX was added at the final concentration of 100 nM and 1 µM, respectively. The incubation medium was renewed every 3 days. The length of hair follicles from the top of hair to the bottom of hair bulb in organ culture was measured with a microscope every third day for 14 days. Hair follicle length was calculated by subtracting the baseline length on day 0 from the length of the same follicle at subsequent evaluation.

### 3.8. Statistical Analysis

Results are expressed as mean ± standard deviation (SD). For statistical analyses, ANOVA with probabilities was performed for both the overall significance and the pairwise comparison (*p*), indicated by asterisks. *p* < 0.05 was considered to be significant.

## 4. Conclusions

In conclusion, we demonstrate that IFN-γ induces catagen-like changes in hDPCs and in hair follicles via inhibition of Wnt/β-catenin signaling, and that MPA stabilizes β-catenin by inhibiting GSK3β leading to increased β-catenin target gene and DP signature gene expression, which may, in part, counteract IFN-γ-induced catagen in hDPCs.
